# Oncocytic Hyperplasia of the Parotid Gland: A Report of a Rare Case

**DOI:** 10.7759/cureus.82795

**Published:** 2025-04-22

**Authors:** Karthika PS, Pola Govardhan Kumar, Raghupathy T, Ankita Swarnkar

**Affiliations:** 1 General Surgery, Shree Balaji Medical College and Hospital, Chennai, IND

**Keywords:** oncocytic hyperplasia, parotidectomy, parotid gland, pleomorphic adenoma, salivary gland tumors

## Abstract

Oncocytic hyperplasia of the parotid gland is a rare, benign condition marked by the proliferation of mitochondria-rich oncocytes, often mimicking neoplasms like pleomorphic adenoma. Its etiology remains unclear but may involve inflammatory triggers, as suggested by an associated granulomatous reaction in this case, possibly linked to prior COVID-19 infection. The condition is exceptionally rare, with limited data on prevalence. Patients typically present with painless parotid swelling, as seen in our 45-year-old male patient, initially suspected to have pleomorphic adenoma based on ultrasound, cytology (Milan category IV A), and contrast-enhanced CT findings. Definitive diagnosis relies on histopathology, which in this case revealed nodular oncocytic hyperplasia with a foreign body granulomatous reaction. Treatment involves surgical excision, such as superficial parotidectomy, to confirm diagnosis and rule out malignancy. This case underscores the diagnostic challenges of oncocytic hyperplasia and highlights the importance of histopathological evaluation to differentiate it from other salivary gland lesions, warranting vigilant monitoring due to its potential, though rare, progression to malignancy.

## Introduction

Salivary gland tumors are heterogeneous groups of neoplasms that can range from benign lesions to malignant cancers [1]. The parotid gland is one of the most frequently affected salivary glands, with pleomorphic adenoma typically presenting as asymptomatic swellings [2]. Oncocytic hyperplasia, characterized by an increase in oncocytic cells, is a less common but important condition that can mimic or precede pleomorphic adenoma [3]. Oncocytic hyperplasia involves the proliferation of oncocyte-epithelial cells with abundant eosinophilic cytoplasm rich in mitochondria [4]. Oncocytic hyperplasia could be considered a premalignant condition since it has the potential for malignant transformation, so early suspicion and recognition of the condition by the healthcare providers, followed by treatment and future follow-up, are crucial [5]. Oncocytic hyperplasia must be differentiated from other salivary gland lesions like pleomorphic adenoma, Warthin tumor (smoking-associated), oncocytoma, mucoepidermoid carcinoma, and chronic sialadenitis, with potential risk factors including chronic inflammation, radiation exposure, aging (40-60 years), prior infections (e.g., COVID-19), and genetic predisposition to mitochondrial dysfunction. A comprehensive diagnostic approach involves clinical evaluation of swelling characteristics, imaging studies (ultrasound/CT/MRI), cytological analysis via FNAC using the Milan system, and definitive histopathological examination through biopsy or surgical excision to confirm diagnosis and exclude malignancy. 

## Case presentation

A 45-year-old man presented with complaints of swelling over the left cheek in front of the ear in the parotid region for six months. The swelling was insidious in onset, initially small, but gradually enlarged to its current size. The patient reported intermittent, low-intensity pain, which was aggravated by jaw movements but had no specific relieving factors. There was no history of purulent discharge from the oral cavity, difficulty in mastication or opening the mouth, jaw locking, salivation, excessive salivation, fever, trauma, mouth ulcers, toothache, or dental caries. Notably, there was no history of other swellings in the neck or on the opposite side of the cheek. The patient denied any significant medical history in the past, was not a smoker, and was nonalcoholic. He did, however, report a recent hospitalization for symptoms related to COVID-19.

**Figure 1:**
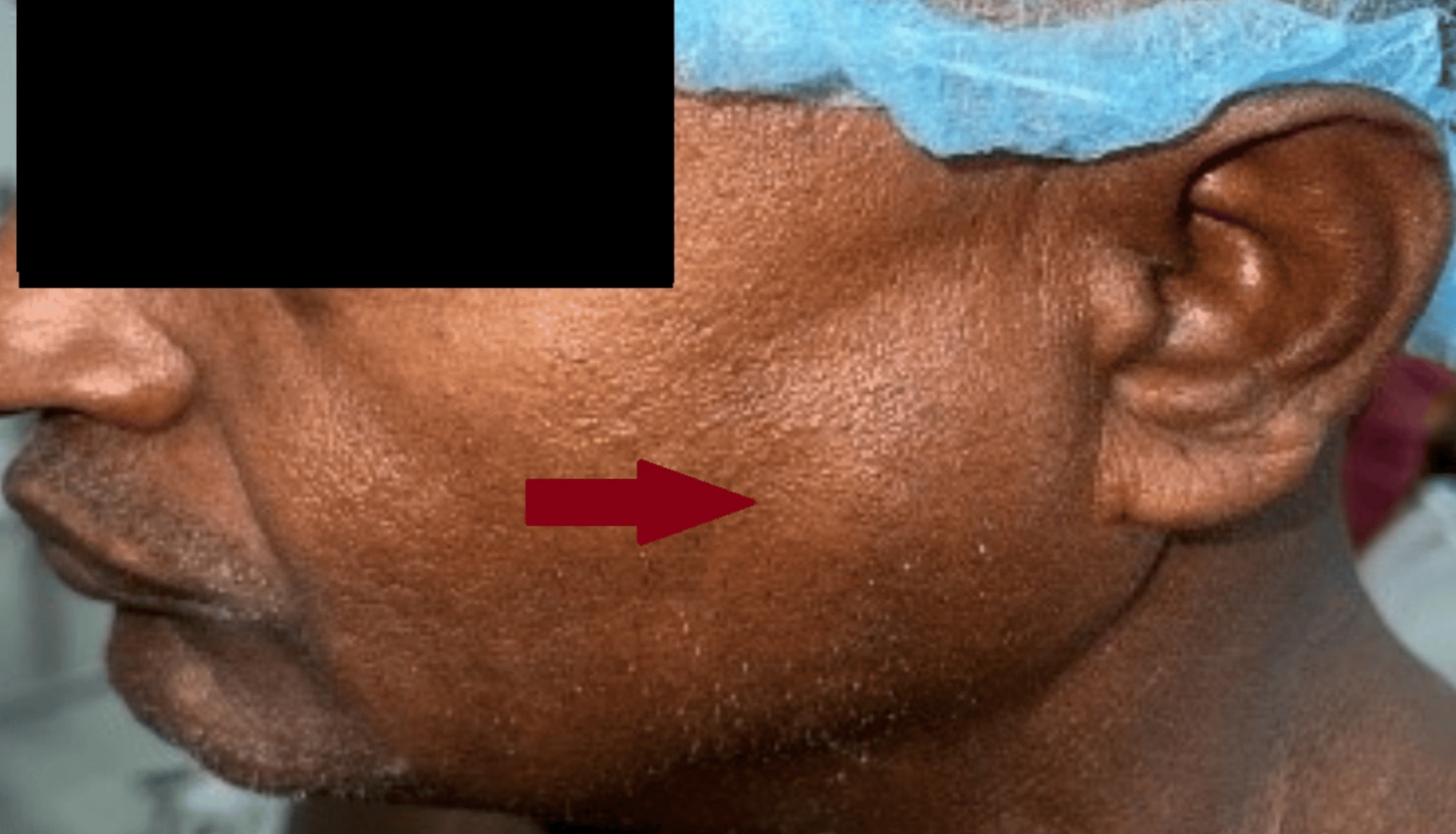
Preoperative image of parotid swelling of size 4x3 cm over the left cheek (lateral view), marked by the red arrow.

**Figure 2:**
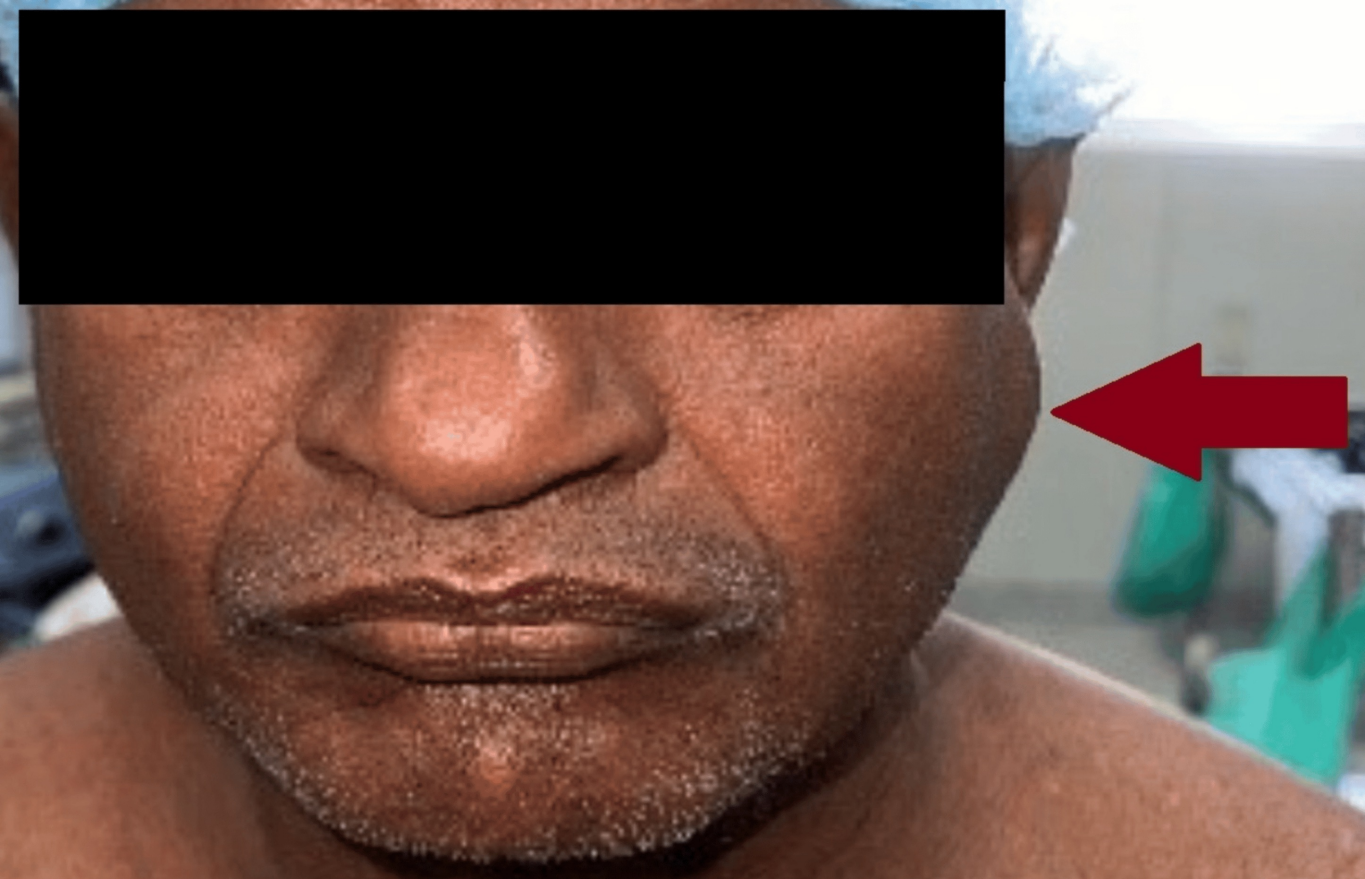
Preoperative image of parotid swelling of size 4x3 cm over the left cheek (anterior view), marked by the red arrow.

Upon local examination, the patient's facial symmetry was disturbed due to the swelling. A firm to hard, oval-shaped mass measuring 4x3 cm was noted in the left parotid region, specifically anterior to the tragus (Figure 1). The swelling extended from 2 cm medial to the tragus and was located 3-4 cm superior to the angle of the mandible, approximately 1 cm from the ramus of the mandible, and 5 cm lateral to the angle of the mouth (Figure 2). No lifting of the left ear lobule was seen. Obliteration of the retromandibular groove was seen. The surface of the swelling appeared smooth, with no skin changes. Swelling became prominent on clenching the teeth and less prominent on opening the mouth. There was no warmth or mild tenderness over the swelling. Importantly, no lymph nodes were palpable. Tongue sensations were normal. Curtain sign was positive. Swelling was firm in consistency, mobile in both directions. The plane of the swelling was deep to deep fascia. Bi-digital palpation of the parotid duct was normal. No lymph nodes were palpable. Another nodule of size 2*1cm with bossellated surface, which was firm in consistency, was freely mobile within the parotid gland. A thorough oral cavity examination revealed no dental caries or abnormalities in the tonsillar region. The parotid duct opening and whatever remained of the oral cavity appeared to be normal. Facial nerve involvement was unremarkable, with the opposite side and other salivary glands appearing intact. Spine/scalp/ ENT examination is normal.

Provisional diagnosis with the above-mentioned history and examination includes a benign tumor of the left parotid gland, most likely a pleomorphic adenoma.

**Figure 3:**
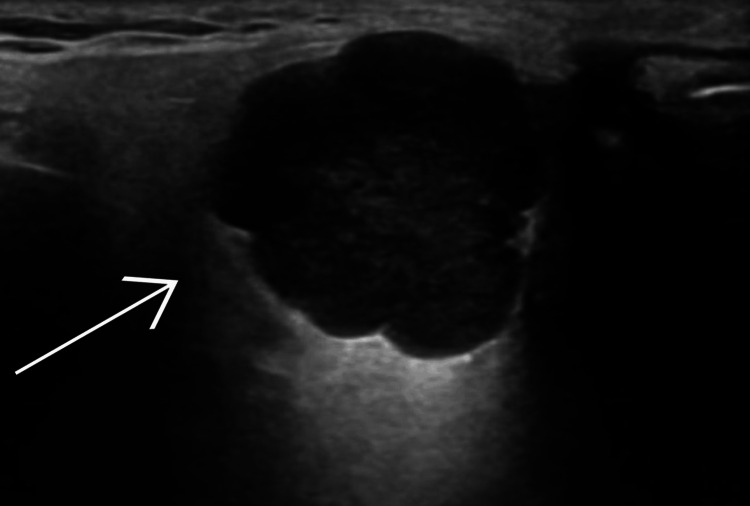
Ultrasonography of superficial swelling of the left parotid gland showing features suggestive of a pleomorphic adenoma marked by a white arrow.

An ultrasound examination of the superficial swelling revealed a well-defined lobular hypoechoic solid-cystic lesion with posterior acoustic enhancement, measuring approximately 1.3 x 0.9 x 0.8 cm in the superficial lobe of the left parotid gland (Figure 3). Color Doppler studies showed no vascularity. Visualized muscles appeared normal. No lymph nodes were seen. These features were suggestive of pleomorphic adenoma.

Cytological analysis indicated features consistent with Milan category IV A, favoring the diagnosis of pleomorphic adenoma (Table 1) [6]. The cytological findings show both epithelial and myoepithelial cells, along with an abundant background of myxoid stroma.

**Table 1:** Milan system for reporting salivary gland cytopathology. Adapted from the table in [6] under Creative Commons Attribution-NonCommercial-ShareAlike License (CC BY-NC-SA).

Diagnostic Category	ROM
I. Non-diagnostic	25%
II. Non-Neoplastic	10%
III. Atypia of undetermined significance (AUS)	20%
IV. Neoplasm	
IVA. Neoplasm: benign	<5%
IVB. Neoplasm: salivary gland Neoplasm of uncertain malignant potential (SUMP)	35%
V. Suspicious for malignancy (SM)	60%
VI. Malignant	90%

**Figure 4:**
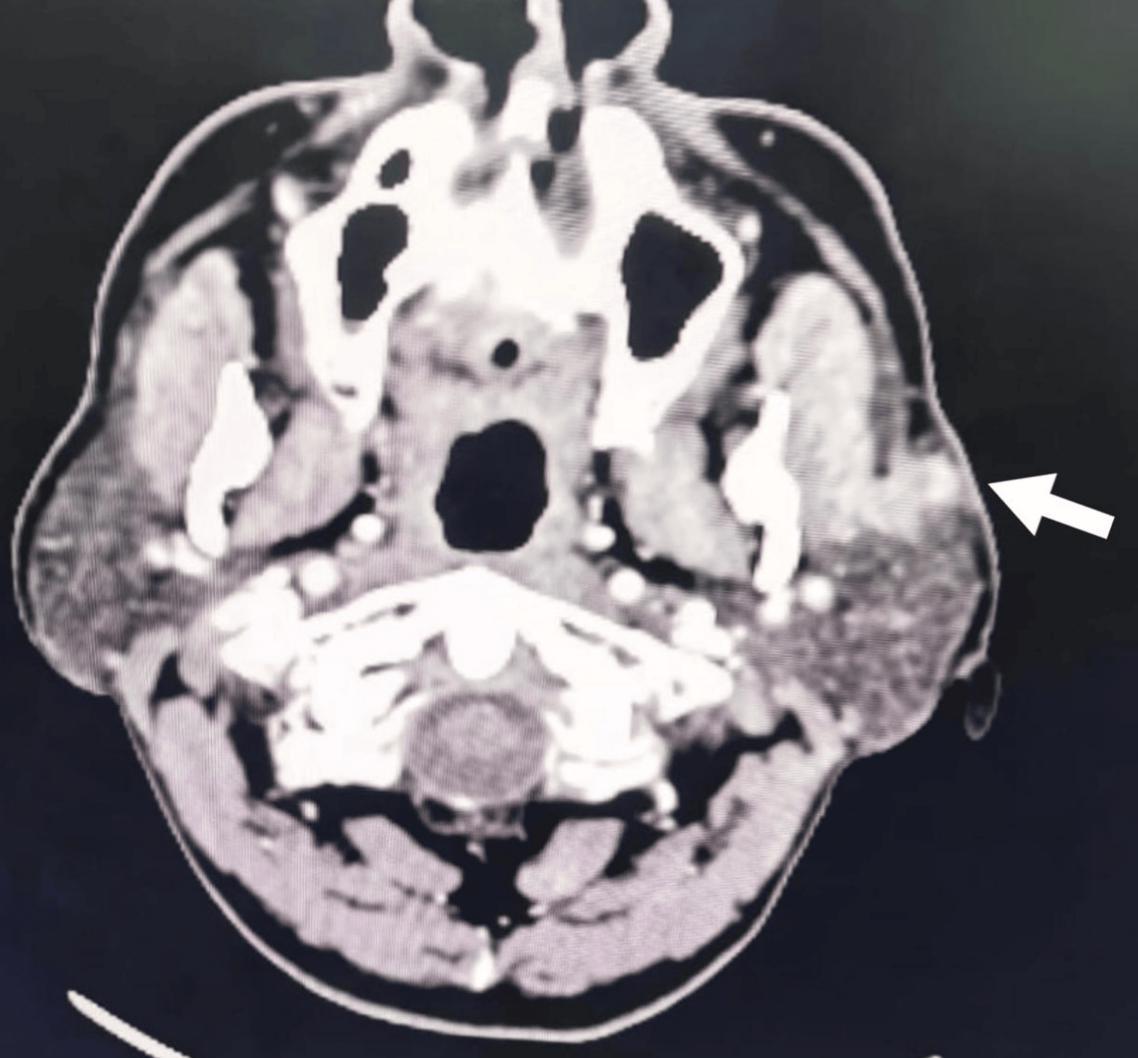
CECT neck showing a heterogenous enhancing soft tissue density lesion of 10*15*12mm size in the superficial lobe of the left parotid gland suggestive of pleomorphic adenoma of the left parotid gland marked by a white arrow. CECT: Contrast-enhanced computed tomography

A contrast-enhanced computed tomography scan of the neck demonstrated a small, well-defined heterogeneous enhancing soft tissue density lesion 10*15*12mm noted in the superficial lobe of the left parotid gland. The lesion was seen abutting the adjacent masseter with minimal surrounding fat stranding. Few subcentimetric lymph nodes were noted in bilateral level IIb. Bones and vessels appeared normal, further supporting the likelihood of a pleomorphic adenoma (Figure 4).

**Figure 5:**
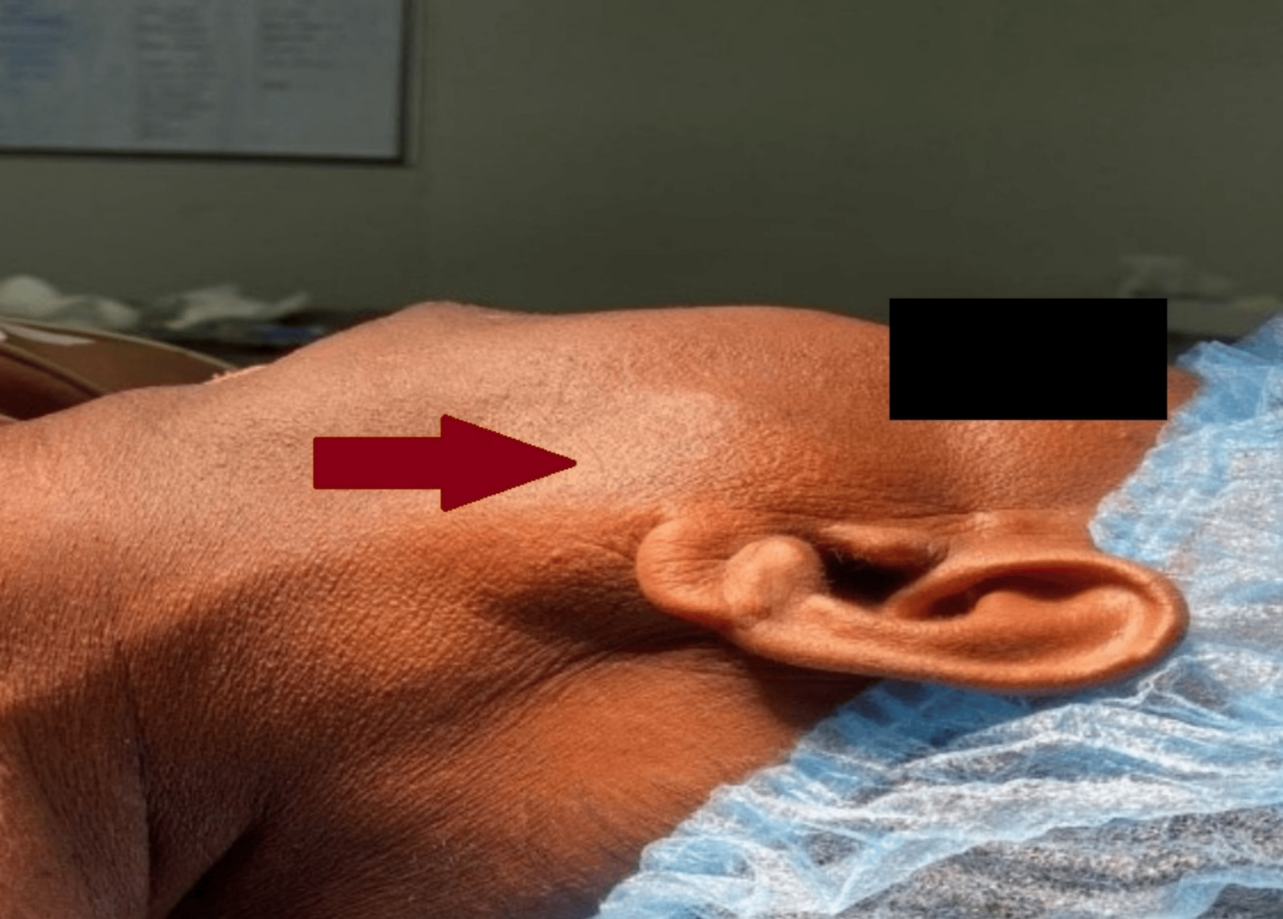
Preoperative image of parotid swelling of size 4x3 cm over the left cheek (on table), marked by the red arrow.

On obtaining anesthesia fitness and required consent, the patient was taken up for superficial parotidectomy (Figure 5).

**Figure 6:**
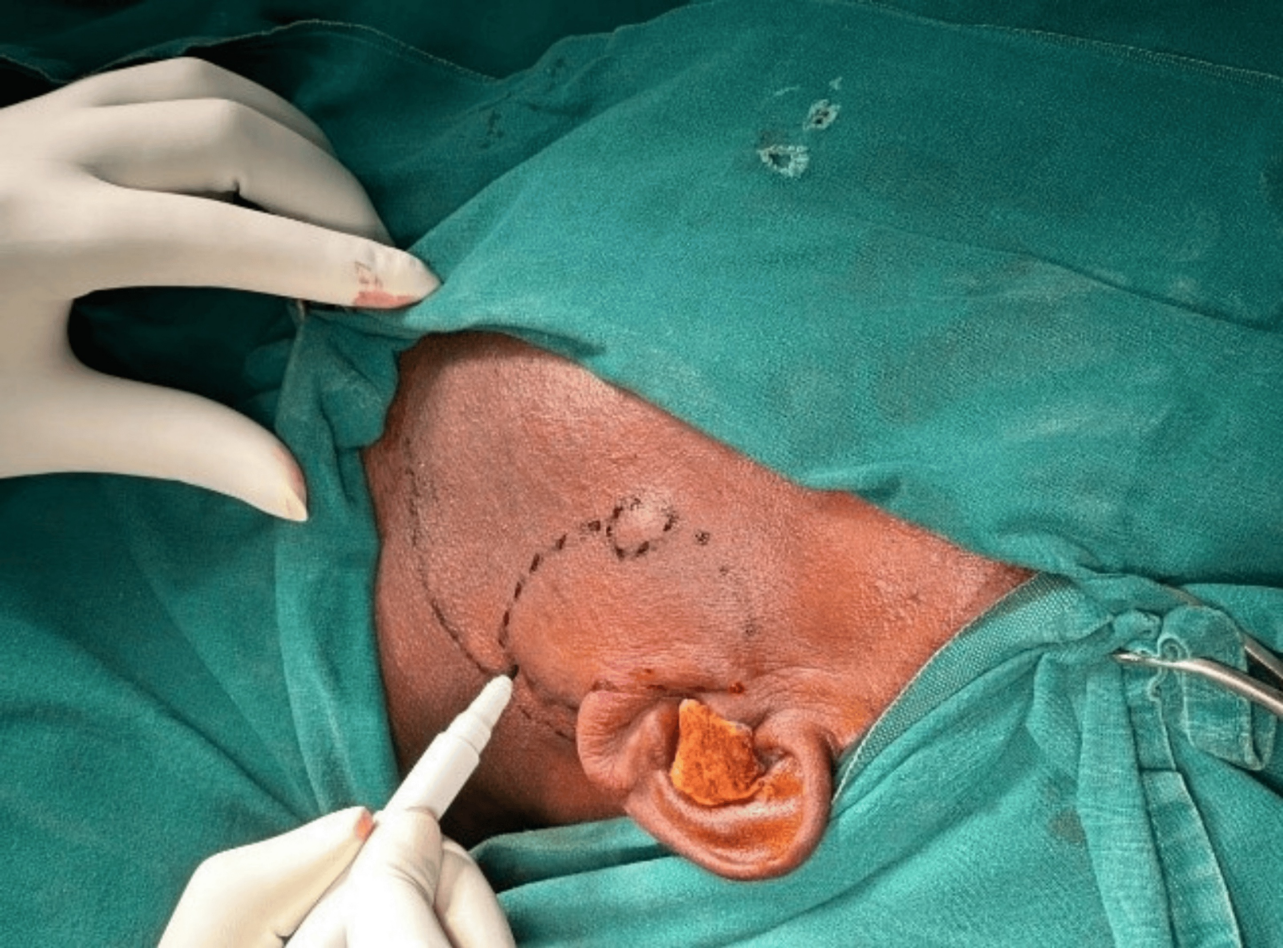
Modified Blair incision marking.

With aseptic precautions, under general anesthesia, the patient was in a supine position with the neck extended and turned to the right side. Parts were painted and draped. Modified Blair incision was done over the left side of the cheek; incision was started above the tragus, extended along the mastoid process, and continued along the sternocleidomastoid, two finger breadths below the angle of mandible along the crease (Figure 6). The incision was deepened beneath the platysma, and the flap was raised until reaching the anterior border of the parotid gland. The skin flap was raised to the Zygomatic Arch, superiorly. The dissection was continued posteriorly along the ear lobe to expose the sternocleidomastoid muscle and mastoid process.

**Figure 7:**
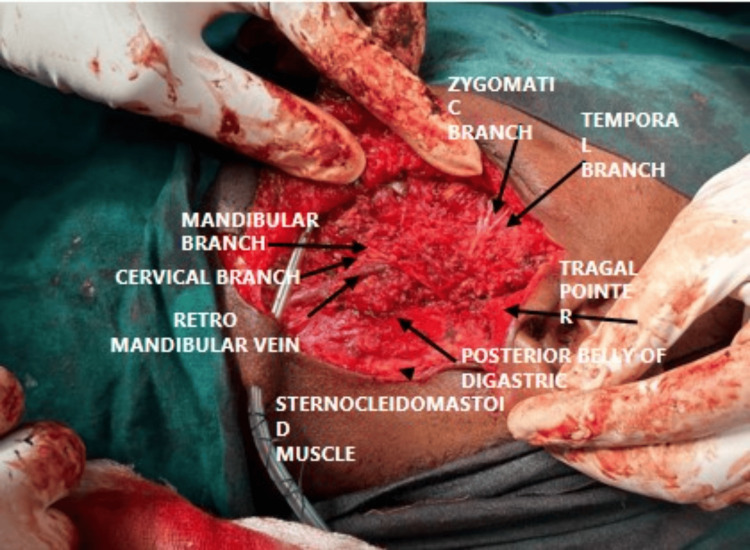
Intraoperative image with labeled structures using black arrows.

**Figure 8:**
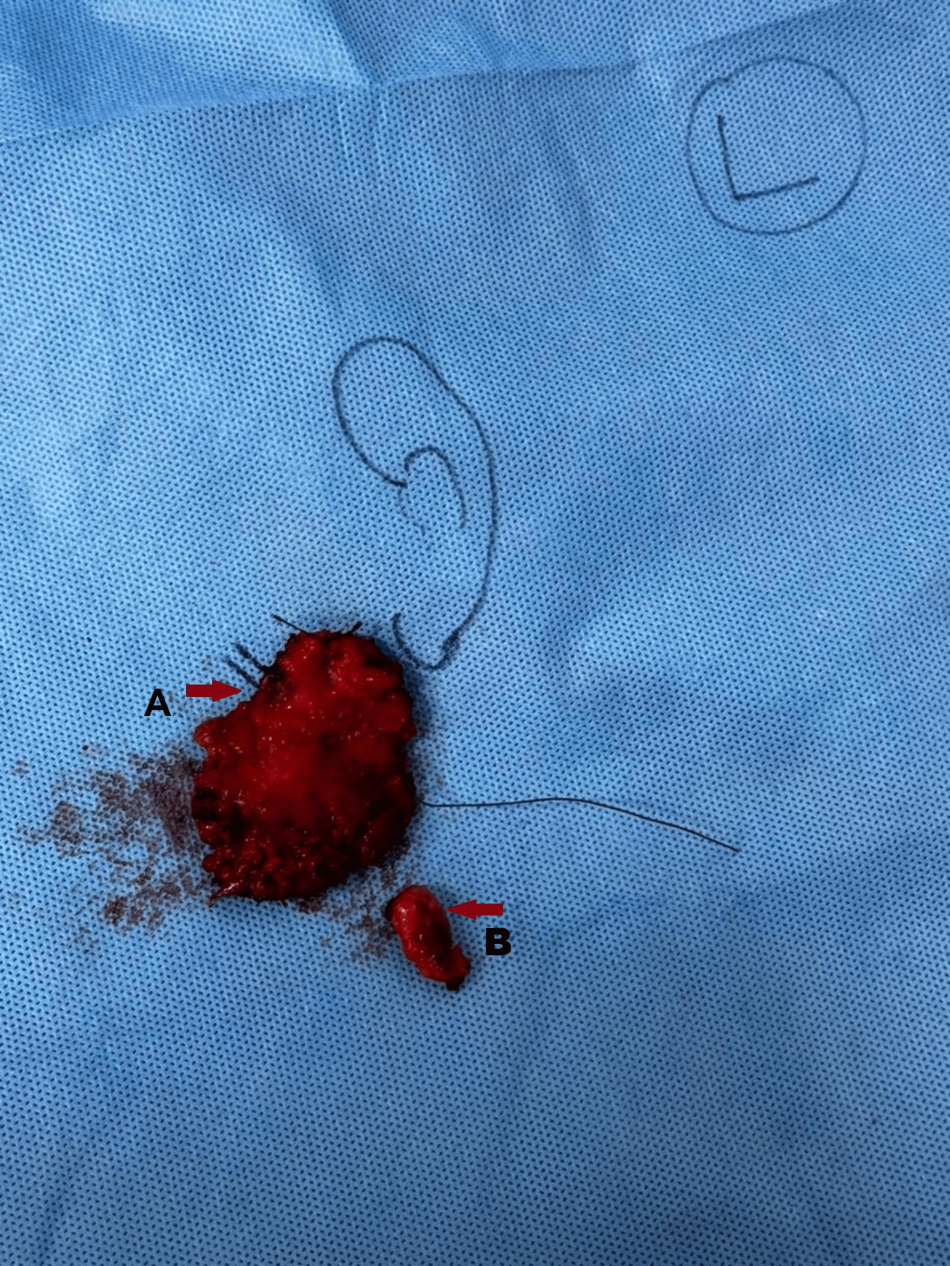
Specimen marking. A: Superficial lobe of parotid marked with a right red arrow and B: Nodule present over the parotid lobe marked with a left red arrow.

A 2*1cm nodular lesion was present over the anterior aspect of parotid gland swelling of size 4*3cm, lateral to the masseter muscle, with evidence of 2 to 3 enlarged parotid lymph nodes present. The greater auricular nerve was exposed, the anterior branch was cut, and the posterior branch was preserved. Dissection was done between the anterior border of the sternocleidomastoid and the posterior border of the parotid gland upward to reach the mastoid process. Sternocleidomastoid retracted laterally to expose the posterior belly of the digastric and stylohyoid muscle. The anterior border of sternocleidomastoid was defined till depth of digastric muscle. The tragal point was identified 1 cm inferomedially, and the facial nerve trunk was localized 1 cm below this landmark, with both the upper temporofacial and lower cervicofacial divisions carefully traced; the parotid gland was then dissected, with intentional sacrifice of the buccal branch due to tumor involvement. The superficial lobe of the parotid gland was fully excised by dissecting along the parotid fascia (Figure 7), removed en bloc, and sent for histopathological examination to confirm the diagnosis (Figure 8). Hemostasis through wound wash was given. A 14-size Romo Vac drain was placed and secured. Subcutaneous closure was done and the skin was approximated. Sterile compression dressing was done.

**Figure 9:**
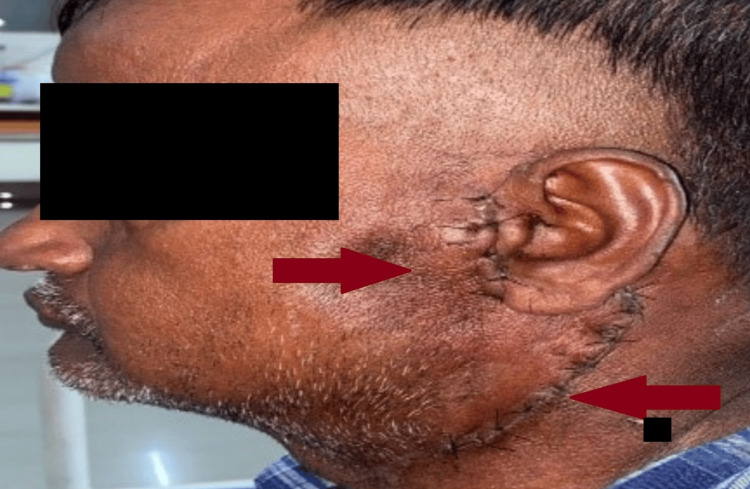
Postoperative image showing a suture line marked with the red arrows.

The patient broke NPO (nil per oral) after six hours. The patient was started on appropriate intravenous fluids, antibiotics, and analgesics. The patient was started on soft solids after 12 hours and had no discomfort while mastication. Postoperatively, mild facial weakness was noticed on the left side, for which he was treated with steroids (Figure 9). Steroids helped with facial asymmetry. The patient's condition improved, and then he was discharged.

After one month, on follow-up, the patient was stable with no complaints (no drooling of saliva, salivation normal, no gustatory sweating, no sensory loss). On examination, the surgical site was healthy, and there was no active discharge or tenderness. 

**Figure 10:**
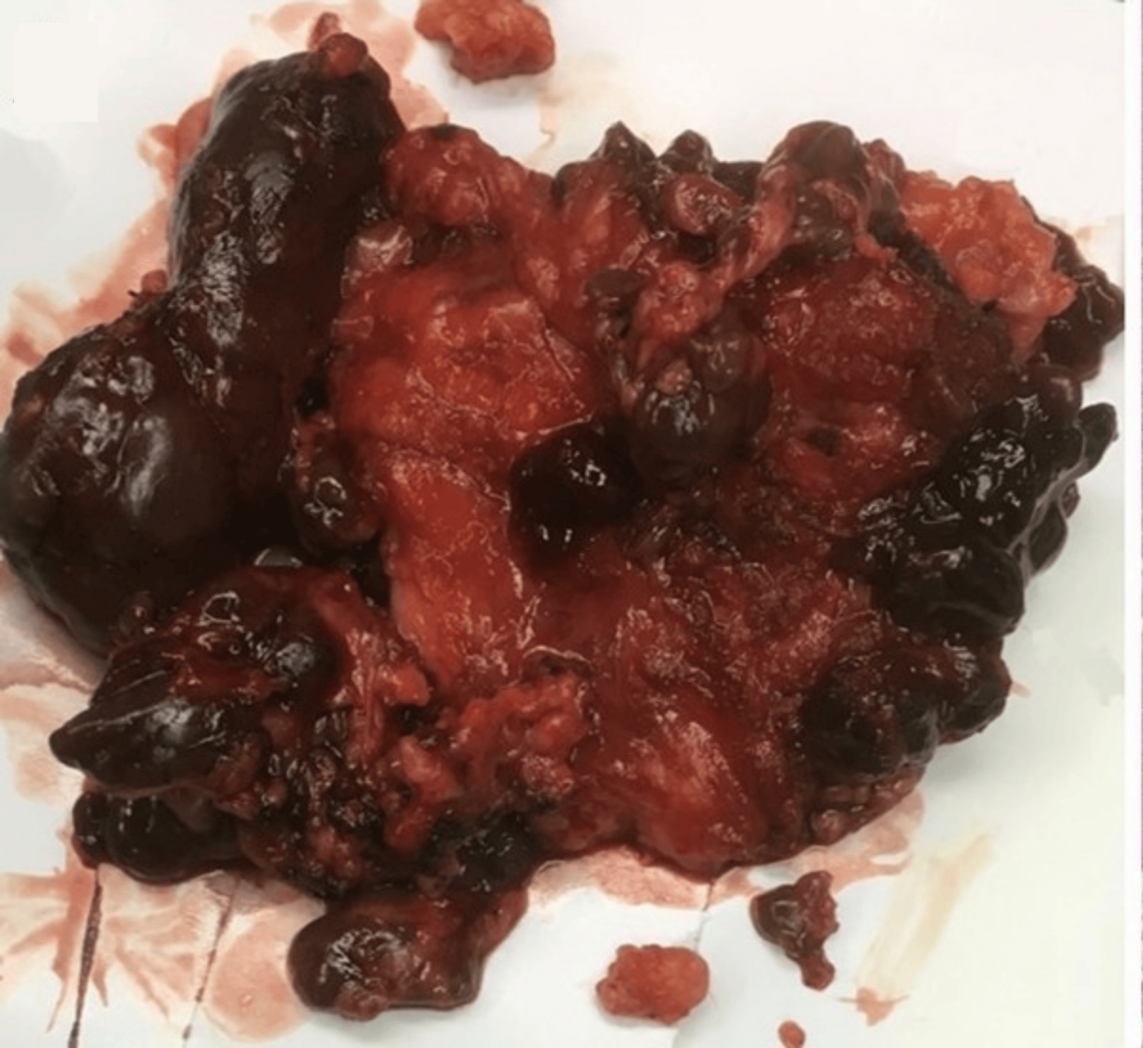
Gross specimen.

The gross pathology shows well-circumscribed, non-encapsulated nodules that are tan, brown with a granular appearance and are usually multifocal (Figure 10).

The histopathological examination report showed nodular oncocytic hyperplasia of the parotid with an adjacent foreign body granulomatous area outside the parotid in the muscle plane.

**Figure 11:**
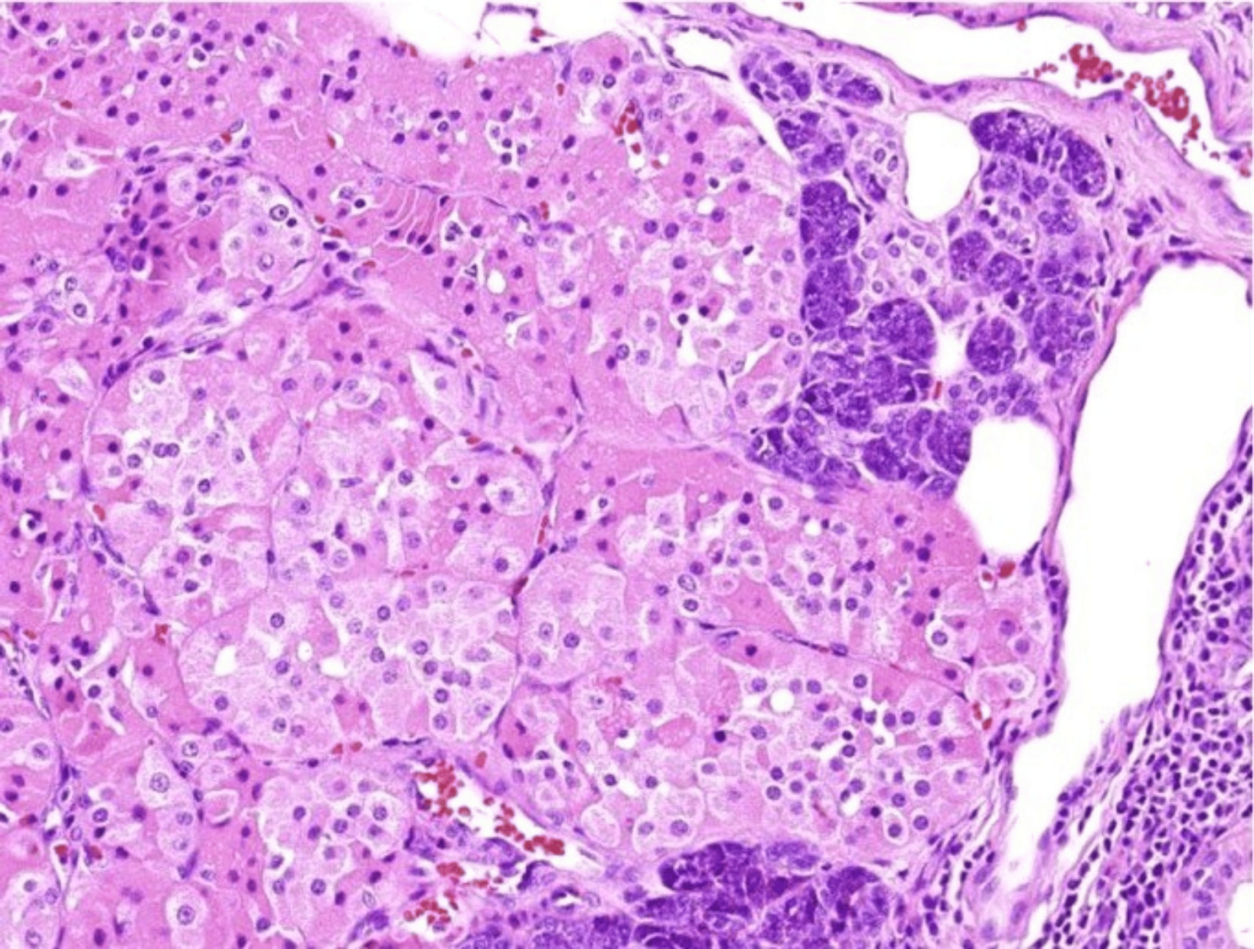
High-power view of nodules in parotid gland parenchyma demonstrating oncocytic cells with granular cytoplasm.

**Figure 12:**
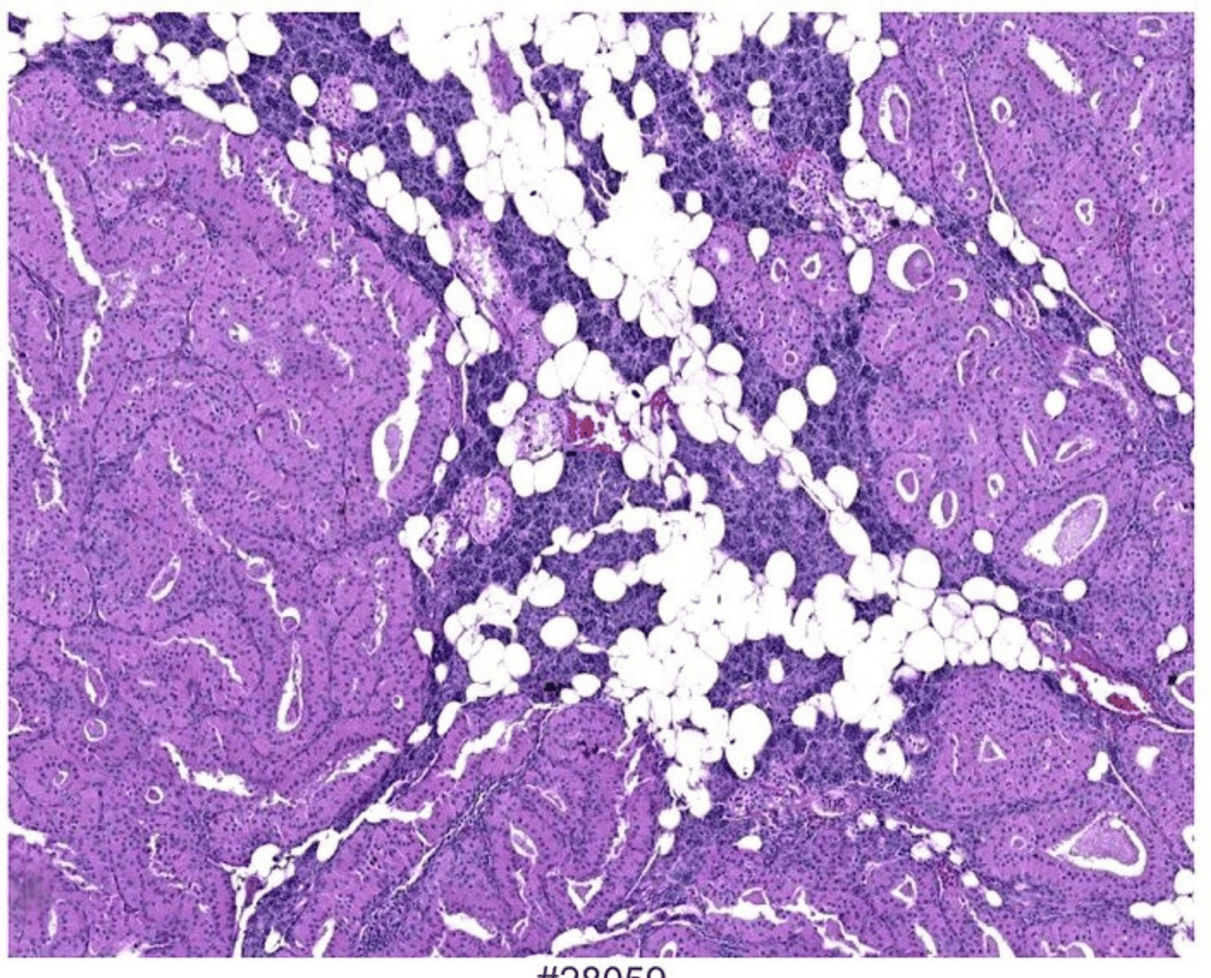
Extensive nodules of oncocytes in the parotid gland parenchyma.

Microscopically, the tumor consists of oncocytic cells with abundant granular eosinophilic cytoplasm (Figure 11) (due to mitochondrial richness), uniform round nuclei, and a nodular architecture separated by fibrous stroma (Figure 12), without significant atypia, mitoses, or necrosis. Histopathology confirmed nodular oncocytic hyperplasia, with an adjacent foreign body granuloma in the muscle plane outside the parotid gland. These findings suggested a possible inflammatory response, which may be linked to the patient’s prior history of COVID-19 or another underlying process.

## Discussion

Oncocytic hyperplasia is a rare entity, particularly in the salivary glands, often diagnosed incidentally during evaluations for other conditions or surgical procedures [7]. It most commonly mimics oncocytomas and Warthin tumors (5-20% of cases) [8], while resemblance to pleomorphic adenomas or malignancies is rarer (<5%). Its clinical significance lies in its potential to progress to malignant tumors, with some cases showing dysplastic features that warrant close monitoring or surgical intervention [9]. The etiology remains unclear, though chronic irritation, inflammation, autoimmune mechanisms, and environmental or infectious triggers (e.g., foreign body granulomatous reactions) have been proposed. Oncocytic hyperplasia may also represent an early stage in pleomorphic adenoma development, as oncocytic changes can occur within established adenomas, underscoring its importance in differential diagnoses for atypical salivary gland masses.

Cellular and genetic changes drive this process: oncocytes (mitochondria-rich cells) proliferate, form nodules, and may undergo metaplasia or dysplasia, contributing to tumor precursors [10]. In pleomorphic adenomas, oncocytes form the epithelial component alongside mesenchymal elements like myxoid or chondroid tissue, supported by genetic alterations [11]. Clinically, these present as painless masses, confirmed via biopsy. Oncocytic hyperplasia, a benign overgrowth of oncocytes, is linked to aging or environmental factors, with rare transformation into oncocytomas due to genetic mutations causing uncontrolled growth. These typically form encapsulated, non-invasive tumors, though malignant progression to oncocytic carcinoma, while rare, is possible via accumulating genetic disruptions. Recurrence occurs in the first three years, necessitating structured follow-up every six months for five years, then yearly thereafter [12]. The literature highlights oncocytic changes in chronic sialadenitis, autoimmune disorders, or post-radiation states, but its rarity demands further research. Histologically, it features oncocyte proliferation with benign architecture and eosinophilic cytoplasm, distinguishable from pleomorphic adenomas, mucoepidermoid carcinomas, or other salivary tumors. Diagnosis combines imaging, clinical evaluation, and histopathology, with surgical excision considered for symptomatic or concerning cases.

The relationship between oncocytic hyperplasia and neoplasia remains a key focus, as it may precede or coexist with tumors like pleomorphic adenomas [13]. While most oncocytomas are benign, their overlap with hyperplasia underscores the need for vigilance in monitoring and differential diagnosis. Chronic inflammatory or environmental triggers may initiate oncocytic changes, but the exact mechanisms require further study. Management balances observation with intervention, prioritizing early detection of malignancy through regular follow-ups, particularly given the potential for recurrence within the first three years. This approach, alongside ongoing research into genetic and environmental drivers, aims to refine diagnostic accuracy and therapeutic strategies for this uncommon but clinically significant condition.

## Conclusions

Oncocytic hyperplasia of the parotid gland is a rare, benign condition that often mimics more common salivary gland tumors like pleomorphic adenoma, posing diagnostic challenges. In our case, a 45-year-old man presented with a painless left parotid swelling initially suspected to be a pleomorphic adenoma on imaging and cytology (Milan IVA), but histopathology confirmed nodular oncocytic hyperplasia with an associated granulomatous reaction, possibly linked to prior COVID-19 infection. Recent advances in molecular pathology have begun to elucidate the role of mitochondrial dysfunction and inflammatory triggers in oncocytic proliferation. Clinically, this case underscores the importance of histopathological confirmation to rule out malignancies and guide management. Treatment remains surgical excision (e.g., superficial parotidectomy) for symptomatic or diagnostically uncertain cases, with intraoperative facial nerve preservation being critical.
